# Angebote zur Prävention von sozialer Isolation und Einsamkeit bei älteren Menschen im ländlichen Raum: Eine gemeindebezogene Bedarfsanalyse

**DOI:** 10.1007/s11553-023-01025-8

**Published:** 2023-03-15

**Authors:** Kathrin Steinbeisser, Sinia Brembeck, Laura Anderle, Christine Boldt

**Affiliations:** 1 Koordinierungsstelle Gesundheitliche Chancengleichheit Bayern, Landeszentrale für Gesundheit in Bayern, Geisenhausenerstraße 18, 81379 München, Deutschland; 2 Fakultät für Angewandte Gesundheitswissenschaften, Technische Hochschule Deggendorf (THD), Dieter-Görlitz-Platz 1, 94469 Deggendorf, Deutschland; 3 Fakultät für angewandte Sozialwissenschaften, Hochschule München, Am Stadtpark 20, 81243 München, Deutschland

**Keywords:** Gesellschaftliche Teilhabe, Gesundheitsförderung, Kommunale Gesundheitsversorgung, Ältere Menschen, Ländlicher Raum, Social participation, Health promotion, Community health services, Elderly, Rural areas

## Abstract

**Hintergrund:**

In ländlichen Regionen sind ältere Menschen besonders mit sozialer Isolation und Einsamkeit sowie deren gesundheitlichen Auswirkungen (z. B. erhöhte Gesamtmortalität, kardiovaskuläre Erkrankungen) konfrontiert. Um diesem zunehmenden Public-Health-Problem entgegenzuwirken, bedarf es Angebote zur Förderung von Sozialkontakten und gesellschaftlicher Teilhabe.

**Fragestellung:**

Liegt ein Bedarf an Präventionsangeboten in Bezug auf soziale Isolation bzw. Einsamkeit vor und wie kann diesem begegnet werden?

**Methoden:**

Eine quantitative, deskriptive Analyse mittels Paper-pencil-Fragebogen wurde von Dezember 2019 bis Januar 2020 durchgeführt, um das (1) Vorliegen von sozialer Isolation und Einsamkeit sowie deren Risikofaktoren, (2) Bedürfnisse und Bedarfe sowie (3) die Eignungsbewertung von und das Interesse an Angeboten zur Prävention von sozialer Isolation und Einsamkeit bei Einwohner/-innen ≥ 65 Jahre einer ländlichen Kommune zu erfassen.

**Ergebnisse:**

Die Rücklaufquote betrug 48,9 % von *N* = 331. In der Studienpopulation lagen verschiedene Risikofaktoren für soziale Isolation und Einsamkeit vor (z. B. Kinderlosigkeit, eingeschränkte Mobilität). Zudem hat fast ein Fünftel der Personen innerhalb von 14 Tagen keinen persönlichen Kontakt zu Menschen aus dem öffentlichen Bereich. Über ein Fünftel gab an, sich „manchmal“ oder „oft“ einsam zu fühlen. Die Angebotsvorschläge „Gottesdienste, Nutzen von kirchlichen Angeboten“, „Ausflüge“, „Informationsveranstaltungen zu verschiedenen Themen“, „Gemeinsame Bewegung/Sport“ und eine „Unterstützungsgruppe, in der man anderen Personen seine Hilfe anbieten und/oder Hilfe bekommen kann“ wurden am häufigsten als geeignet sowie interessant beurteilt.

**Schlussfolgerungen:**

Die Ergebnisse zeigen einen hohen Bedarf, Bedürfnisse und Interesse an Angeboten zur Prävention von sozialer Isolation und Einsamkeit auf. Angebote sollten die spezifischen Bedarfe und Bedürfnisse (z. B. geringe finanzielle Mittel, eingeschränkte Mobilität) älterer Menschen berücksichtigen.

**Zusatzmaterial online:**

Zusätzliche Informationen sind in der Online-Version dieses Artikels (10.1007/s11553-023-01025-8) enthalten.

## Abstract (en)

**Background:**

Older people living in rural areas are at high risk of social isolation and loneliness, as well as their associated health effects (e.g., increased all-cause mortality, cardiovascular diseases). To address this increasing public health problem, offers to promote and strengthen social contacts and participation are necessary.

**Objectives:**

Is there a need for preventive offers regarding social isolation or loneliness, and how can this be met?

**Methods:**

A quantitative, descriptive analysis using a paper–pencil questionnaire was conducted from December 2019 to January 2020 to analyze (1) the presence of social isolation and loneliness, as well as its risk factors, (2) needs and wants, and (3) the assessment of suitability and interest in offerings to prevent social isolation and loneliness among residents ≥ 65 years in a rural community.

**Results:**

The response rate was 48.9% out of *N* = 331. Various risk factors for social isolation and loneliness (e.g., childlessness, restricted mobility) were present in the study population. Furthermore, almost a fifth did not have any personal contact with people from the public sector within the last 14 days. Over a fifth said they felt lonely “sometimes” or “often”. The proposed offerings “worship services, use of church-based measures”, “excursions”, “informational events on various topics”, “joint exercise/sports” and a “support group in which one can offer one’s help to other people and/or receive help” were most frequently rated as suitable and interesting.

**Conclusions:**

The results show high demand, needs, and interest in offers which prevent social isolation and loneliness. Services should consider the specific needs (e.g., affordability) and wants (e.g., community actions) of older people.

## Hintergrund

### Soziale Isolation und Einsamkeit im Alter

Gemäß den Ergebnissen des Deutschen Alterssurvey (DEAS; [10]) liegt das Risiko sozialer Isolation in Deutschland bei etwa 12 % für 65-Jährige und steigt bis zum 90. Lebensjahr auf etwa 22 % an. Soziale Isolation wird als ein quantitativer Mangel von sozialen Kontakten verstanden, unabhängig von deren subjektiv bewerteter Bedeutung für das Individuum [7, 10].

**Abb. 1:**
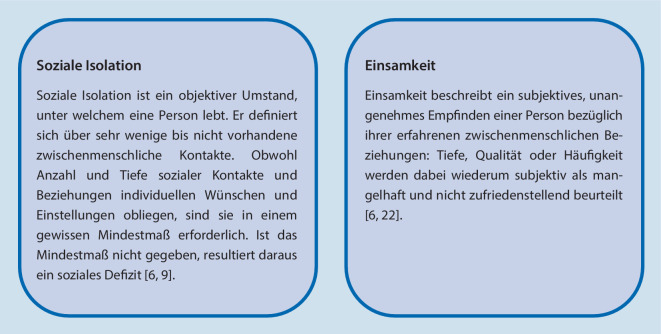
Definition von sozialer Isolation [6, 9] und Einsamkeit [6, 22]

Der sozialen Isolation steht, wie in Abb. 1 dargestellt, die Einsamkeit gegenüber. Auch bezüglich dieser – einem subjektiven, unangenehmen Empfinden einer Person bezüglich ihrer erfahrenen zwischenmenschlichen Beziehungen – wird eine steigende Tendenz im Alter, insbesondere ab dem 70. Lebensjahr, angenommen [6, 10, 22]. Etwa 8 % der 65-Jährigen weisen ein Risiko auf, von Einsamkeit betroffen zu sein. Ab einem Alter von 90 Jahren tritt Einsamkeit bei etwa 22 % der Personen auf [10, 13].

### Gesundheitliche Auswirkungen durch soziale Isolation und Einsamkeit

Soziale Isolation und Einsamkeit stehen in Zusammenhang mit negativen gesundheitlichen Auswirkungen [3, 16]. Soziale Isolation ist assoziiert mit einer erhöhten Gesamtmortalität, dessen Assoziation selbst nach Ausschluss von Suizid fortbesteht. Des Weiteren stehen eine erhöhte Anzahl von Einweisungen in die Notaufnahme sowie Krankenhausaufenthalte nachweislich in Verbindung mit sozialer Isolation bei älteren Menschen. Auch gesundheitsgefährdende Verhaltensweisen, wie etwa ein geringes Maß an körperlicher Aktivität, liegen bei sozial isolierten oder einsamen Personen häufiger vor als bei Menschen, die sozial stärker eingebunden sind [16]. Konkrete Erkrankungen, die mit sozialer Isolation und Einsamkeit einhergehen können, sind Schmerzen im unteren Rücken, die chronisch-obstruktive Lungenerkrankung (COPD), allgemeine kognitive Einschränkungen bis hin zu demenziellen Erkrankungen sowie einer Vielzahl kardiovaskulärer, onkologischer, neurologischer und psychiatrischer Krankheitsbilder [3].

### Angebote zur Prävention von sozialer Isolation

Um sozialer Isolation entgegenzuwirken, bedarf es geeigneter Angebote und Ansätze, welche eine Steigerung der Anzahl sozialer Interaktionen begünstigen. Zu diesen zählen beispielsweise soziale Unterstützungsangebote, (Weiter‑)Bildungsangebote, Sport‑/Bewegungsgruppen sowie infrastrukturelle Maßnahmen in der Wohnumgebung. Besonders erfolgversprechend sind Angebote, wenn die Teilnehmenden deren Umsetzung aktiv mitgestalten (partizipativer Ansatz) und auf deren Bedürfnisse abgestimmt sind (zielgruppenspezifischer Ansatz; [5, 14]).

### Angebote zur Prävention von Einsamkeit

Um dem Erleben von Einsamkeit erfolgreich entgegenwirken zu können, bedarf es Angebote und Ansätze, die den betroffenen Personen individuelle und subjektiv passgenaue Lösungsansätze anbieten [8]. Hierunter fallen beispielsweise Angebote zum Zeitvertreib in einer Gruppe oder der persönliche Kontakt mit einer anderen Person, gesellschafts- und gemeindebezogene Angebote, Diskussionsgruppen, Kurse zum Erlernen neuer Fähigkeiten und Gruppen für emotionale und informationelle Unterstützung. Konkrete Beispiele für Aktivitäten zur Prävention von Einsamkeit sind gemeinsames Tanzen, Musizieren, Malen, gemeinschaftliche Gartenarbeit, Ausflüge oder Sport- und Bewegungsangebote. Das Anbieten von Informationszugängen sowie Angebote von niedrigschwelligen Anlaufstellen sind weitere Möglichkeiten, die Menschen als Wege aus ihrer Einsamkeit dienen können [21]**.**

### Relevanz und Fragestellungen der Studie

Der Bedarf des Ausbaus von Angeboten zur Prävention von Einsamkeit und sozialer Isolation gewinnt insbesondere auch aufgrund der Erfahrungen der SARS-CoV-2-Pandemie („severe acute respiratory syndrome coronavirus 2“) an Bedeutung. Im Vergleich zu 2019 ist ein Gefühl von Einsamkeit bei Älteren vermehrt festzustellen. Während 2019 etwa 8 % der 60- bis 69-Jährigen und etwa 11 % der ≥ 70-Jährigen sich mindestens wöchentlich einsam fühlten, waren es 2020 etwa 35 % bzw. 27 % [17]. Zudem ist infolge der Reduktion von Angeboten während der Pandemie eine verminderte Inanspruchnahme sozialer Aktivitäten durch Ältere zu erkennen [26]. In einer ländlichen Kommune wurde bereits vor der SARS-CoV-2-Pandemie eine rückläufige Teilnahme am öffentlichen und sozialen Leben älterer Einwohner/‑innen festgestellt. Politische Entscheidungsträger und Gesundheitsfachpersonen vermuteten unter den älteren Einwohner/‑innen ein erhöhtes Risiko für soziale Isolation und Einsamkeit. Ferner nahmen sie wahr, dass an bisherigen Angeboten zur Förderung der sozialen Einbindung, beispielsweise an Ausflügen in die nähere Umgebung oder der Senior/-innensportgruppe, stets nur eine feste Personengruppe teilnahm. Gleichzeitig waren bestimmte Personen seltener in der Kommune präsent. Aufgrund der negativen gesundheitlichen Auswirkungen von sozialer Isolation und Einsamkeit [3, 16] sollten deshalb zielgruppen-, bedarfs- und bedürfnisgerechte Präventionsangebote für Einwohner/-innen ≥ 65 Jahre in der Kommune geschaffen werden. Folgende Forschungsfragen sollen mithilfe der Bedarfsanalyse beantwortet werden:

- Liegen soziale Isolation, Einsamkeit und/oder Risikofaktoren von sozialer Isolation bzw. Einsamkeit bei Einwohner/-innen ≥ 65 Jahre vor?
- Welche Bedürfnisse und welchen Bedarf äußern Einwohner/-innen ≥ 65 Jahre an Angeboten zur Prävention von sozialer Isolation und Einsamkeit?
- Welche Angebote zur Prävention von sozialer Isolation und Einsamkeit werden von Einwohner/-innen ≥ 65 Jahre als geeignet und interessant eingeschätzt?

## Methodik

### Modellkommune und Studienpopulation

Die Studie fand in einer ländlich gelegenen, bayerischen Kommune (*n* ≤ 2000 Einwohner/-innen) statt. In der Kommune, jedoch nicht in allen Teilorten, befinden sich Geschäfte der Nahversorgung (z. B. kleiner Supermarkt, Metzgerei, Bäckerei), eine Poststelle, Friseursalons und Handwerksunternehmen. Einrichtungen, wie z. B. eine Finanzbank, wurden geschlossen. Der öffentliche Personennahverkehr wird mithilfe von Busverbindungen in die nähere Umgebung, welche sich auf punktuelle Zeiten des Berufs- und Schulverkehrs beschränken, sowie einem Ruftaxi, das bei Bedarf von den Bürger/-innen selbst angefordert werden kann, bewerkstelligt. In der Kommune leben 331 Personen im Alter ≥ 65 Jahren (Stand: 2019). Davon sind ungefähr 35 % ledig, geschieden oder verwitwet. Alle Personen ≥ 65 Jahre und wohnhaft in der Kommune, wurden für die Bedarfserhebung eingeschlossen.

### Erhebungsinstrument

Als zielgruppengerechtes Erhebungsinstrument wurde ein aus 29 Fragen bestehender quantitativer, teilstandardisierter, Paper-pencil-Fragebogen erstellt (s. Anhang 1 und 2). Der Fragebogen beinhaltet offene, halboffene und geschlossene Fragen. Als Grundlage dienten Fragen aus den Erhebungen „Leben und Wohnen in Bad Sassendorf“ [19], „Seniorenbefragung Ahaus 2015“ [18], „Leben im Alter in Linkenheim-Hochstetten“ [9], „Health and lifestyles of people aged 50 and over. Self-completion questionnaire“ [1], „Gesundheit in Deutschland aktuell, GEDA“ [15], „Germany. ISSP-2017 – Social Networks and Social Resources Questionnaire“ [12] und „Leben in Europa 2015. Personenfragebogen“ [24]. Die Konzeption erfolgte in Zusammenarbeit mit Vertreter/-innen der Kommune sowie Expert/-innen aus Pflegewissenschaft, Epidemiologie, Gesundheitsförderung und Prävention. Dabei wurde die Handhabbarkeit der älteren Zielgruppe fokussiert (z. B. große Schrift, verständliche Ausfüllhilfe). Ein Pretest wurde mit sieben repräsentativen Zielgruppenvertreter/-innen durchgeführt. Darauf basierend erfolgten in geringem Maße Adaptationen.

### Erhebungsmethoden und Datenauswertung

Im Dezember 2019 wurden an die zuvor definierte Zielgruppe Fragebögen mittels persönlicher Übergabe durch eine Pflegestudierende und geschulte Multiplikator/-innen (Vertreter/-innen der Gemeindeverwaltung, Gesundheits- und Seniorenbeauftragte der Kommune, freiwillige Helfer/-innen) verteilt. Dabei erhielten die Einwohner/-innen Informationen über die Befragung sowie zur Freiwilligkeit und Anonymität der Teilnahme sowohl schriftlich im Fragebogen als auch mündlich im Rahmen einer persönlichen Übergabe.

Zur Abgabe der Fragebögen befanden sich Sammelboxen bei den verteilenden Personen sowie im Rathaus, in der Bäckerei und Metzgerei. Außerdem konnten die Teilnehmenden eine Abholung des Fragebogens durch Personal der Gemeindeverwaltung anfordern. Zur Erhöhung der Teilnahmemotivation hatten die Befragten die Möglichkeit, an einem Gewinnspiel teilzunehmen. Die Kommune verloste 20 Wertgutscheine in Höhe von jeweils 10 €, welche im Rahmen von ausgewählten künftigen Veranstaltungen für Speisen und Getränke eingelöst werden konnten. Um die Anonymität sicherzustellen, wurden die Teilnehmenden darum gebeten, den Fragebogen und die Teilnahmeerklärung für das Gewinnspiel in die ausgehändigten, separaten und eindeutig beschrifteten Briefumschläge zu geben.

Die Daten wurden deskriptiv anhand von Mittelwerten und Relationen zwischen Risikofaktoren und Zielvariablen analysiert und mittels SPSS, Version 26.0 (IBM Corp., Armonk, NY, USA) ausgewertet.

## Ergebnisse

Die Antworten auf einzelne Fragen erfolgten nicht konsistent von allen Befragten („missings“). Zudem bestand der Fragebogen z. T. aus offenen Fragen sowie Fragen mit der Option auf Mehrfachnennung. Diese Aspekte führen dazu, dass die Grundgesamtheit einzelner Fragen von der Gesamtanzahl von *N* = 162 teilweise abweicht.

### Stichprobe

Am Stichtag des 29.01.2020, dem finalen Abgabetermin, lagen 162 Fragebögen vor. Dies stellt eine Rücklaufquote von 48,9 % dar. Die Altersverteilung lag zwischen 65 und 99 Jahren, im Durchschnitt bei 75 Jahren (Standardabweichung: 6,8 Jahre, *N* = 149). Die Geschlechterverteilung lag bei 51,6 % (*n* = 82) weiblichen und 48,4 % (*n* = 77) männlichen von insgesamt 159 Teilnehmenden, die diese Frage beantworteten. Ein niedriger sozioökonomischer Status war bei 13,6 % der Befragten festzustellen (s. Tab. 1).

**Tab. 1:** Soziodemografische Charakteristika der Studienpopulation

	*N*^a^	*n*	Anteil (%)
*Alter*^b^	149		
65–74 Jahre		83	55,7
75–84 Jahre		52	34,9
85–94 Jahre		9	8,7
≥ 95 Jahre		1	0,7
*Geschlecht*	159		
Weiblich		82	51,6
Männlich		77	48,4
Divers		0	0,0
*Kinder (Ref.: nein)*	156		
Ja		143	91,7
*Bildungsjahre (Ref.: ≥* *10 Jahre)*^b^	134		
< 10 Jahre		89	66,4
*Monatliches Haushaltsnettoeinkommen*	132		
< 900 €		52	39,4
900–1299 €		29	22,0
1300–1499 €		20	15,2
1500–1999 €		15	11,4
2000–2599 €		8	6,1
≥ 2600 €		8	6,1
*Erwerbsstatus*^c^	157		
Vollzeit erwerbstätig		1	0,6
Teilzeit erwerbstätig		3	1,9
Hausfrau/-mann		38	24,2
Altersteilzeit		1	0,6
Vorruhestand		3	1,9
Ruhestand/Rente		145	92,4
Minijob/450-€-Job		9	5,7
Arbeitssuchend		0	0,0
Sonstiges		0	0,0
*Berufsbezeichnung*^b, c^	95		
Landwirt/-in		12	12,6
Arbeiter/-in		12	12,6
Angestellte/-r		50	52,6
Beamt/-in		5	5,3
Selbstständige/-r		7	7,4
Hausfrau		13	13,7
*Bezug staatlicher Leistungen (z.* *B. Rente, Grundsicherung) (Ref.: nein)*^c^	148		
Ja		139	93,9
*Haushaltsgröße*^b^* (Ref.: Mehrpersonenhaushalt)*	137		
Einpersonenhaushalt		32	23,4
*Mitbewohner/-innen im selben Haus*^c^	157		
(Ehe‑)Partner/-in		109	69,4
Kind(er)		83	52,9
Enkelkind(er)		49	31,2
Geschwister		3	1,9
Eltern		0	0,0
Andere Verwandte		1	0,6
Freund/-innen		0	0,0
Nachbar/-innen, Bekannte		2	1,3
Haustier(e)		11	7,0
Sonstige		4	2,5
Niemand		11	7,0
*Art des Wohnhauses*	158		
Einfamilienhaus		79	50,0
Zweifamilienhaus		73	46,2
Mehrfamilienhaus		5	3,2
Seniorenheim		0	0,0
Sonstiges		1	0,6

^a^Abweichungen zur Gesamtanzahl der Studienpopulation (N = 162) ergeben sich aufgrund fehlender Angaben zu jeweiligem Item

^b^Offene Frage

^c^Bei diesem Item waren Mehrfachnennungen möglich

### Vorliegen von sozialer Isolation und Einsamkeit sowie Risikofaktoren

Aufgrund der Ähnlichkeit sozialer Isolation und Einsamkeit in Bezug auf deren Vorliegen und Risikofaktoren werden diese nachfolgend zusammenfassend dargestellt [4, 20]. Zur Bestimmung des Vorliegens von sozialer Isolation und Einsamkeit wurde erhoben, zu welchen Personen aus dem Familien- und Bekanntenkreis (z. B. eigene Kinder, Vereinskolleg/-innen, Nachbar/-innen) sowie dem öffentlichen Bereich (z. B. Pflegekräfte eines ambulanten Dienstes, Friseur/-in, Verkäufer/-in) „14-tägig normalerweise mindestens einmal Kontakt“ bestand. 1,3 % (*N* = 154) der Teilnehmenden gaben an, dass sie „14-tägig normalerweise“ zu Niemandem aus dem Familien- und Bekanntenkreis persönlichen Kontakt hatten. Keinen Kontakt zu Personen aus dem öffentlichen Bereich hatten 14-tägig 17,1 % (*N* = 123) der Teilnehmenden. Alle Teilnehmenden hatten „14-tägig normalerweise mindestens einmal Kontakt“ zu Personen aus dem Familien- und Bekanntenkreis oder aus dem öffentlichen Bereich. Der Anteil an Personen, die sich „manchmal“ oder „oft“ einsam fühlen, lag bei 20,8 % (*N* = 159). Sich „selten oder nie“ einsam zu fühlen, gaben 79,2 % (*N* = 159) der Teilnehmenden an.

Bei Betrachtung der Risikofaktoren zeigen die Ergebnisse, dass 8,3 % (*N* = 156) der Teilnehmenden kinderlos sind und 23,4 % (*N*= 137) gaben an, allein zu leben. Weniger als 10 Bildungsjahre wiesen 66,4 % (*N* = 134) auf. Als zuletzt ausgeübten Beruf gaben 38,9 % (*N* = 95) Landwirt/-in, Arbeiter/-in oder Hausfrau an. Hinsichtlich des monatlichen Haushaltsnettoeinkommens zeigte sich bei > 40 % der Teilnehmenden ein Unterschreiten der monatlichen Armutsgefährdungsschwelle, die 2019 für ≥ 65-Jährige bei 1021 € lag [11, 25]. Ihren derzeitigen allgemeinen Gesundheitszustand bewerteten 46,8 % (*N* = 158) der teilnehmenden Personen als „mittelmäßig“, „schlecht“ oder „sehr schlecht“. „Im Alltag auf Geh- oder Mobilitätshilfen angewiesen zu sein“, gaben 20,3 % (*N* = 158) der Teilnehmenden an.

Treffen mit Familienangehörigen, die nicht im selben Haushalt mit den Befragten leben, fanden bei 70,8 % (*N* = 154) der Teilnehmenden „wöchentlich“ oder „(mehrmals) täglich“ statt. 24,0 % (*N* = 154) sahen diese Personengruppe „monatlich oder seltener“. Bei 5,2 % (*N* = 154) fanden die Treffen entweder „nie“ statt oder es erfolgte die Angabe „ich habe keine“ Familienangehörigen. Von den Teilnehmenden verließen 90,3 % (*N* = 155) ihr Haus beziehungsweise ihre Wohnung „täglich“ oder „mehrmals pro Woche, aber nicht täglich“. Insgesamt 3,9 % (*N* = 155) verließen ihr Haus beziehungsweise ihre Wohnung „seltener als einmal pro Woche“. Die Aktivitäten, welche die Teilnehmenden als Anlässe nannten, um ihr Zuhause zu verlassen, lagen dabei vorrangig in alltäglichen Erledigungen, aber auch in der Interaktion mit Anderen. „Einkaufen“ (68,0 %, *N* = 128) und „Arzttermine“ (39,8 %, *N* = 128) waren die häufigsten genannten Gründe, um das Zuhause zu verlassen. Darauf folgten „Spazierengehen“ (19,5 %, *N* = 128), „Besuche von Freund/-innen, Bekannten und Verwandten“ (18,8 %, *N* = 128) und „zur Kirche gehen“ (15,6 %, *N* = 128). Gegebenheiten oder Situationen, welche im Alltag das Verlassen des Zuhauses verhindern und damit Einschränkungen in der Verrichtung alltäglicher Erledigungen darstellen, nannten 33,9 % (*N* = 59) der Teilnehmenden. Am häufigsten wurden „eingeschränkte körperliche Mobilität“ (18,6 %, *N* = 59), „reduzierter Gesundheitszustand“ (10,2 %, *n* = 59) sowie „pflegebedürftige/‑r Ehepartner/‑in“ (3,4 %, *N* = 59) genannt (s. Tab. 1 und 2).

**Tab. 2:** Vorliegen von sozialer Isolation und Einsamkeit sowie Risikofaktoren

	*N*^a^	*n*	Anteil (%)
*Allgemeiner Gesundheitszustand*	158		
Sehr gut		6	3,8
Gut		78	49,4
Mittelmäßig		57	36,1
Schlecht		14	8,9
Sehr schlecht		3	1,9
*Verwendung von Geh- oder Mobilitätshilfen*^c^* (Ref.: Nein)*	158		
Ja		32	20,3
*Häufigkeit des Verlassens des Zuhauses*	155		
Täglich		91	58,7
Mehrmals pro Woche, aber nicht täglich		49	31,6
Einmal pro Woche, aber nicht häufiger		9	5,8
Seltener als einmal pro Woche		6	3,9
*Gründe des Verlassens des Zuhauses*^b, c^	128		
Einkaufen		87	68,0
Arzttermine		51	39,8
Besuche von Freunden, Bekannten und Verwandten		24	18,8
Spazierengehen		25	19,5
Zur Kirche gehen		20	15,6
Sport und Bewegung in einer (festen) Gruppe/Verein		14	10,9
Gartenarbeit		10	7,8
Fahrradfahren		8	6,3
Wald‑/Holzarbeit		8	6,3
Friseurbesuch		6	4,7
Ehrenamt		3	2,3
Arbeit		5	3,9
Arbeiten rund ums Haus		6	4,7
Aufsuchen anderer gesundheitsbezogener Angebote		7	5,5
Versorgung von Tieren		5	3,9
Allgemeine Besorgungen		4	3,1
Landwirtschaftliche Tätigkeiten		4	3,1
Seniorengaststätte		5	3,9
Friedhofsbesuch		4	3,1
Vereinsaktivitäten		3	2,3
Sonstige Unternehmungen		4	3,1
Auswärts essen		3	2,3
Veranstaltungen besuchen		3	2,3
Wandern		2	1,6
Treffen von bekannten in öffentlichem rahmen		2	1,6
Fürsorgeaufgaben im familiären Umfeld		1	0,8
Sonstige Bewegung		1	0,8
Sonstiges		3	2,3
Nie		2	1,6
*Hindernisse beim Verlassen des Zuhauses*^b, c^	59		
Keine		39	66,1
Eingeschränkte körperliche Mobilität		11	18,6
Reduzierter Gesundheitszustand		6	10,2
Pflegebedürftige/r Ehepartner/-in		2	3,4
Fehlende öffentliche Verkehrsanbindung		1	1,7
Winterliche Bedingungen		2	3,4
Persönliche Einstellung		1	1,7
*Freizeitgestaltung*^c^	157		
Sportliche Betätigung		40	25,5
Spazierengehen		100	63,7
Kurse besuchen/weiterbilden		2	1,3
Gartenarbeit		102	65,0
Ehrenamtliche Tätigkeit		23	14,7
Busreisen/Tagesausflüge		31	19,8
Treffen mit Freund/-innen/Bekannten		61	38,9
Besuch kultureller Veranstaltungen		27	17,2
Vereinstätigkeit		38	24,2
Zeitung/Bücher lesen		113	72,0
Telefonieren		61	38,9
Hausarbeit		100	63,7
Betreuung von Familienangehörigen		40	25,5
Handwerkliche Tätigkeiten		52	33,1
Basteln		16	10,2
Grabpflege		54	34,4
Sonstiges		8	5,1
*Häufigkeit des Kontakts mit Familienangehörigen außerhalb des Zuhauses*	154		
(Mehrmals) täglich		37	24,0
Wöchentlich		72	46,8
Monatlich oder seltener		37	24,0
Nie		5	3,3
Keine Familienangehörigen		3	2,0
*Kontaktpersonen im Familien- und Bekanntenkreis*^c, d^	154		
Ehe- oder Lebenspartner/-in		58	37,7
Eigene Kinder		129	83,7
Enkelkinder oder andere Verwandte		98	63,6
Freund/-innen, Bekannte, Vereinskolleg/-innen		79	51,3
Nachbar/-innen		73	47,4
Sonstige		6	3,9
Niemand		2	1,3
*Kontaktpersonen im öffentlichen Bereich*^c, d^	123		
Arzt/Ärztin		41	33,3
Pflegekräfte/Mitarbeitende eines ambulanten Dienstes		13	10,6
Pfarrer/-in oder Mitglieder einer Kirchengemeinde		20	16,3
Friseur/-in		18	14,6
Verkäufer/-in		60	48,8
Sonstige		5	4,1
Niemand		21	17,1
*Subjektives Einsamkeitsgefühl*	159		
Selten oder nie		126	79,2
Manchmal		30	18,9
Oft		3	1,9

^a^Abweichungen zur Gesamtanzahl der Studienpopulation (N = 162) ergeben sich aufgrund fehlender Angaben zu jeweiligem Item

^b^Offene Frage

^c^Bei diesem Item waren Mehrfachnennungen möglich

^d^Mit diesen Personen bestand innerhalb von 14 Tagen mindestens einmal Kontakt

### Bedürfnis und Bedarf nach Präventionsangeboten in Bezug auf soziale Isolation und Einsamkeit

Bezüglich der geäußerten Bedürfnisse nach Präventionsangeboten bestätigten 61,2 % (*N *= 147) der Teilnehmenden durch die Angaben „stimme voll zu“ bis „stimme eher zu“ ihren Wunsch, häufiger „unter Leute kommen“ zu wollen. 38,8 % (*N* = 147) hingegen stimmten dem „eher nicht“ bis „gar nicht“ zu. Insgesamt 9,3 % (*N* = 162) der Teilnehmenden äußerten sich hierzu nicht. Bezüglich des Bedarfs an Präventionsangeboten waren 76,8 % (*N* = 151) der Teilnehmenden der Meinung, dass es vor Ort genügend gesellschaftliche und gesellige Angebote für sie gäbe. Dem widersprachen 23,2 % (*N* = 151; s. Tab. 3).

**Tab. 3:** Bedürfnis und Bedarf nach Präventionsangeboten in Bezug auf soziale Isolation und Einsamkeit

	*N*^a^	*n*	Anteil (%)
*Wunsch nach mehr Kontakten (Ref.: stimme eher nicht zu/stimme gar nicht zu)*	147		
		90	61,2
*Ausreichende Angebotsauswahl (Ref.: nein)*	151		
Ja		116	76,8
*Zufriedenheit (sehr zufrieden/zufrieden) mit aufgeführten Angeboten (Ref.: unzufrieden/sehr unzufrieden)*			
Einkaufsmöglichkeiten	146	131	89,7
Freizeitangebote	124	95	76,6
Kulturangebote	104	64	61,5
Ärztliche Versorgung	135	55	40,7
Versorgung durch Apotheken	133	68	51,1
Notdienst der Ärzte	124	71	57,3
Notdienst der Apotheken	123	59	48,0
Öffentliche Verkehrsmittel	125	47	37,6
Gaststätten	142	112	78,9
Öffnungszeiten des Rathauses	149	141	94,6
Bisherige Angebote der Gemeinde	120	104	86,7
Sonstiges	2	1	50,0

^a^Abweichungen zur Gesamtanzahl der Studienpopulation (N = 162) ergeben sich aufgrund fehlender Angaben zu jeweiligem Item

### Eignungsbewertung von und Interesse an Präventionsangeboten

Die Angebotsvorschläge, welche die Teilnehmenden am geeignetsten einschätzten, um sie beim Pflegen und Knüpfen von zwischenmenschlichen Kontakten zu unterstützen, sind „Ausflüge“ (76,4 %, *N* = 110), „Gottesdienste, Nutzen kirchlicher Angebote“ (72,7 %, *N* = 110), „Informationsveranstaltungen zu verschiedenen Themen“ (77,0 %, *N* = 100), eine „Unterstützungsgruppe, in der man anderen Personen seine Hilfe anbieten und/oder Hilfe bekommen kann“ (69,9 %, *N* = 103) sowie „Gemeinsame Bewegung/Sport“ (62,5 %, *N* = 112). Keine Möglichkeit für sich an den vorgeschlagenen Angeboten teilzunehmen, sahen 2 Teilnehmende.

Die Angebotsvorschläge, welche die Teilnehmenden am meisten interessierten, sind „Gottesdienstbesuche, Nutzen kirchlicher Angebote“ (68,3 %, *N* = 101), „Ausflüge“ (68,7 %, *N* = 99) sowie „Informationsveranstaltungen zu verschiedenen Themen“ (69,6 %, *N* = 92; s. Tab. 4).

**Tab. 4:** Eignungsbewertung von und Interesse an Präventionsangeboten

	*N*^a^	*n*	Anteil (%)
*Eignungsbewertung (stimme zu/stimme eher zu) von aufgeführten Angeboten (Ref.: stimme eher nicht zu/stimme nicht zu)*			
Essen in Gesellschaft	110	60	54,6
Gemeinsames Spazierengehen	110	60	54,6
Gemeinsames Musizieren, Singen	105	34	32,4
Tanzabende	107	33	30,8
Weiterbildungskurse	101	52	51,5
Unterstützungsgruppe	103	72	69,9
Gemeinsame Bewegung/Sport	112	70	62,5
Gesellschaftsspiele spielen	109	53	48,6
Gemeinsames Kochen	103	27	26,2
Ausflüge	110	84	76,4
Informationsveranstaltungen	100	77	77,0
Bastel‑/Malangebote	100	26	26,0
Gartenarbeit in Gesellschaft	97	21	21,7
Handwerkliche Tätigkeiten in Gesellschaft	100	35	35,0
Gottesdienstbesuche, Nutzen kirchlicher Angebote	110	80	72,7
Filmabende	101	49	48,5
Sonstige	5	2	40,0
*Interesse (stimme zu/stimme eher zu) an aufgeführten Angeboten (Ref.: stimme eher nicht zu/stimme nicht zu)*			
Essen in Gesellschaft	103	49	47,6
Gemeinsames Spazierengehen	101	51	50,5
Gemeinsames Musizieren, Singen	94	23	24,5
Tanzabende	87	22	25,3
Weiterbildungskurse	99	45	45,5
Unterstützungsgruppe	94	54	57,4
Gemeinsame Bewegung/Sport	101	58	57,4
Gesellschaftsspiele spielen	99	46	46,5
Gemeinsames Kochen	92	20	21,7
Ausflüge	99	68	68,7
Informationsveranstaltungen	92	64	69,6
Bastel‑/Malangebote	84	20	23,8
Gartenarbeit in Gesellschaft	88	15	17,1
Handwerkliche Tätigkeiten in Gesellschaft	92	27	29,4
Gottesdienstbesuche, Nutzen kirchlicher Angebote	101	69	68,3
Filmabende	84	35	41,7
Sonstige	2	1	50,0

^a^Abweichungen zur Gesamtanzahl der Studienpopulation (N = 162) ergeben sich aufgrund fehlender Angaben zu jeweiligem Item

## Diskussion

Die vorliegende Bedarfsanalyse gibt einen Überblick über das Vorliegen von sozialer Isolation und Einsamkeit sowie deren Risikofaktoren, über Bedürfnisse und den Bedarf sowie über die Eignung von Angeboten zur Prävention von sozialer Isolation und Einsamkeit bei Einwohner/-innen ≥ 65 Jahre einer ländlichen Kommune.

Die Ergebnisse zusammenfassend betrachtet, ergeben sich Hinweise für ein Risiko bzw. das Vorliegen von sozialer Isolation und Einsamkeit bei den Einwohner/-innen der ländlichen Kommune. Ein Vergleich mit bereits bestehenden Untersuchungsergebnissen bestätigt diese Annahme: In der vorliegenden Befragung hat ein bestimmter Anteil an Personen innerhalb von 14 Tagen keinen persönlichen Kontakt zu Menschen aus ihrem Familien- und Bekanntenkreis (1,3 %) oder dem öffentlichen Bereich (17,1 %). Damit scheinen die befragten Personen etwas seltener von sozialer Isolation betroffen zu sein als die 12–22 % Befragten des DEAS [10].

Allerdings liegt der Anteil der Personen, die in der Bedarfsanalyse angaben, sich „manchmal“ oder „oft“ einsam zu fühlen mit 20,8 % deutlich über dem im DEAS [10] kalkulierten Einsamkeitsrisiko von 8–11 %. Wichtig zu erwähnen ist, dass die Prävalenz der Personen, die sich einsam fühlen, nur zu einem bestimmten Teil erhoben werden kann (z. B. aufgrund von Scham; [2]). Daher ist zu vermuten, dass der Anteil an Personen, die sich einsam fühlen, höher als der in dieser Studie erfasst ist. Schulungen von Personen gängiger Anlaufstellen (z. B. Ärzt/‑innen, Friseur/‑innen) zur Identifikation von einsamen Personen oder von Personen mit Risikofaktoren für Einsamkeit können dabei unterstützen, weitere Personen dieser vulnerablen Gruppe zu identifizieren und folglich für präventive Maßnahmen zu berücksichtigen.

Weitaus mehr Teilnehmende wiesen Risikofaktoren für soziale Isolation und Einsamkeit auf, die über die Einschlusskriterien, dem Alter und dem Wohnort, hinausgehen. Diese waren Merkmale wie alleinlebend oder Mobilitätseinschränkungen. Hinsichtlich des monatlichen Haushaltsnettoeinkommens zeigt sich bei > 40 % der Teilnehmenden ein Unterschreiten der monatlichen Armutsgefährdungsschwelle, die 2019 für ≥ 65-Jährige bei 1021 € lag [10, 24]. Betrachtet man die vorliegende Bildung, die Einkommensverhältnisse und die gängigen Berufe, wird erkennbar, dass einige der Teilnehmenden auch den Risikofaktor des geringen sozioökonomischen Status aufweisen (vgl. [23]). Aufgrund der Betroffenheits- und Risikolage kann deshalb ein Bedarf an entsprechenden Präventionsmaßnahmen angenommen werden.

Angebote, die zwischenmenschliche Begegnungen ermöglichen und deren Anzahl erhöhen, sind einem großen Anteil der Befragten ein Bedürfnis, wenngleich dies nicht für alle Teilnehmenden gleichermaßen zutrifft. Die Vorstellungen der Teilnehmenden darüber, welche Angebote sich ihrer Ansicht nach dafür eignen würden, um sie beim Knüpfen und Pflegen zwischenmenschlicher Kontakte zu unterstützen, decken sich neben den Äußerungen zum Teilnahmeinteresse an bestimmten Angebotsvorschlägen auch mit Erkenntnissen und Empfehlungen bisheriger Forschungsarbeiten [5, 21]**. **Neben der Eignungsbewertung ist eine Betrachtung des Interesses der Teilnehmenden essenziell, um einen Zielgruppenbezug im Rahmen der Good Practice Ansätze herzustellen [14]. Aufgrund der Veränderungen durch die SARS-CoV-2-Pandemie bedarf es weiterer wissenschaftlicher Untersuchungen zum Interesse an neuartigen Angeboten, wie etwa digitalen Austauschtreffen [26]. Bei Abwägungen zur praktischen Umsetzung der Präventionsmaßnahme sollte unabhängig von der Wahl einer konkreten Präventionsmaßnahme den spezifischen Anforderungen der Zielgruppe Beachtung geschenkt werden. Einschränkungen in der körperlichen und räumlichen Mobilität, etwa eine Angewiesenheit auf Gehhilfen oder die beschränkte Verfügbarkeit öffentlicher Verkehrsmittel, erschweren es vielen Senior/-innen bereits im Alltag, außer Haus zu gehen. Außerdem verfügen die meisten Befragten über ein geringes Einkommen. Die Ergebnisse führen zum Schluss, dass infrastrukturelle Barrieren und höhere Kostenaufwendungen für die potenziellen Teilnehmenden als starke Hemmnisfaktoren einzuschätzen sind und bei der Planung und Umsetzung von Maßnahmen besonders beachtet werden müssen.

### Stärken und Limitationen

Im Rahmen der Bedarfsanalyse hat sich das hohe Engagement von Seiten des Bürgermeisters, der Pflegestudierenden und relevanter Multiplikator/-innen der Kommune als bedeutsamer Gelingensfaktor bewährt. Die Vernetzung von Akteur/-innen unterschiedlichster Disziplinen und das große Engagement dieser sind in Verbindung mit partizipativen Arbeitsweisen auch für die folgende Planung nachhaltiger Ansätze vielversprechend. Die hohe Rücklaufquote der vorliegenden Befragung ist eine Stärke dieser Studie, die vermutlich auf das hohe Engagement sowie die vernetzte Arbeit in der Kommune zurückzuführen ist. Zu den weiteren begünstigenden Faktoren zählen die niedrigschwellige Rückgabemöglichkeit sowie der Abholservice der Fragebögen. Die vorliegende Bedarfsanalyse und deren Ergebnisse können einen Anreiz für andere Kommunen darstellen, eine Analyse dieser Art eigens durchzuführen.

Limitationen dieser Studie liegen in der rein deskriptiven Analyse der Daten, sodass sich die Aussagen auf Trends beschränken.

## Fazit für die Praxis

- Eine gemeindebezogene Befragung von Senior/-innen erweist sich als hilfreiches Instrument zur Bedarfsabfrage und als eine Grundlage zur Planung passgenauer Präventionsmaßnahmen, um der sozialen Isolation und subjektiv empfundenen Einsamkeit entgegenzuwirken.
- Das Interesse an Präventionsangeboten in Bezug auf Einsamkeit und soziale Isolation liegt besonders auf Veranstaltungen, welche der gesellschaftlichen Teilhabe dienen.
- Eine Berücksichtigung von Personen in schwierigen Lebenslagen und Einschränkungen beim Bewältigen des Alltags ist elementar und kann unter anderem durch niedrigschwellige, kostengünstige Angebote und Unterstützungsmöglichkeiten zur erleichterten Teilnahme gelingen.
- Die Förderung des Pflegens von Kontakten kann auch durch einen erweiterten Einbezug von Personen aus dem öffentlichen Bereich erfolgen, da diese für viele Befragte Teil des Alltags und Grund zum Verlassen des Hauses sind.

## Supplementary Information

Fragebogen zur Bedarfserhebung in der Kommune

Anschreiben zum Fragebogen
