# Supplementary material for: Offers to prevent social isolation and loneliness in older people living in rural areas: A community-based survey of needs
Source: Pravent Gesundh. 2023 Mar 15:1–10. [Article in German] Online ahead of print. doi: 10.1007/s11553-023-01025-8 (PMC10015520; doi:10.1007/s11553-023-01025-8)
Supplement: Supplementary file 1 — Fragebogen zur Bedarfserhebung in der Kommune [file 11553_2023_1025_MOESM1_ESM.pdf]

Möchten Sie Ihren Alltag in [redacted] verbessern?

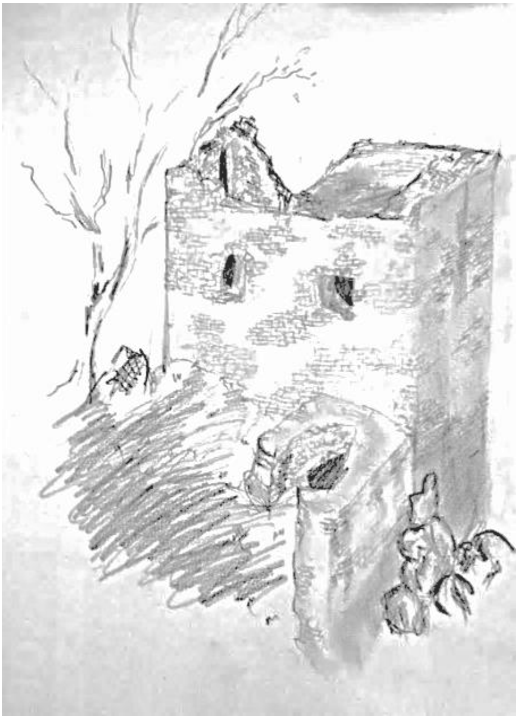

Befragung

der Einwohnerinnen und Einwohner

der Gemeinde [redacted] ab 65 Jahren

Sehr geehrte Damen und Herren,

wir freuen uns über Ihre Teilnahme an der Befragung. Auf der rechten Seite finden Sie noch einige Hinweise zum Ausfüllen des Fragebogens.

Ihre Teilnahme ist freiwillig! Alle von Ihnen angegebenen Daten werden anonym und vertraulich behandelt!

Wenn Sie den Fragebogen ausgefüllt haben, stecken Sie ihn bitte in den beigefügten Briefumschlag (Beschriftung „FRAGEBOGEN“). Sie können den Fragebogen entweder bei einer der im Anschreiben genannten Kontaktpersonen abgeben, ihn in eine der Sammelboxen werfen, die in den genannten Geschäften und auf der Informationsveranstaltung am 29.01.2020 in [redacted] bereitstehen, oder in den Briefkasten des Rathauses [redacted] einwerfen.

Abgabeschluss ist der 29.01.2020!

Falls Sie Fragen haben oder Unterstützung beim Ausfüllen wünschen, wenden Sie sich gerne an das Rathaus [redacted].

Mit freundlichen Grüßen

[redacted] – *Bürgermeister*

[redacted] – *Gesundheitsbotschafterin*

[redacted] – *Studentin; Durchführung und Auswertung der Befragung*

Hinweise zum Ausfüllen des Fragebogens

<table>
<tr><td colspan="2">Bitte beantworten Sie die Fragen möglichst der Reihe nach. Lassen Sie Fragen aus, die Sie nicht beantworten möchten oder können.</td></tr>
<tr><td>☐ ja ☒ nein</td><td>Bitte kreuzen Sie das Kästchen bei der für Sie zutreffenden Antwort deutlich an.</td></tr>
<tr><td>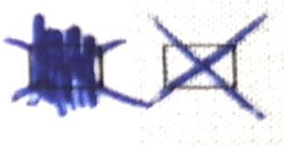</td><td>Um Antworten zu korrigieren, schwärzen Sie das versehentlich gesetzte Kreuz deutlich und setzen ein neues.</td></tr>
<tr><td>Bitte nur ein Kreuz!</td><td>Bitte entscheiden Sie sich für <u>eine</u> Antwort.</td></tr>
<tr><td>Mehrfachnennungen möglich!</td><td>Bitte wählen Sie alle Antworten aus, die auf Sie zutreffenden.</td></tr>
<tr><td>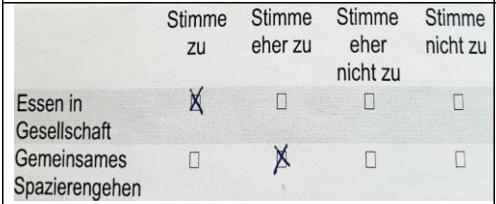
<table>
<tr><th></th><th>Stimme zu</th><th>Stimme eher zu</th><th>Stimme eher nicht zu</th><th>Stimme nicht zu</th></tr>
<tr><td>Essen in Gesellschaft</td><td>☒</td><td>☐</td><td>☐</td><td>☐</td></tr>
<tr><td>Gemeinsames Spazierengehen</td><td>☐</td><td>☒</td><td>☐</td><td>☐</td></tr>
</table></td><td>Finden Sie die Antwort-möglichkeiten in einer Tabelle mit einer Skala, dann setzen Sie <u>pro Zeile</u> bitte nur ein Kreuz.</td></tr>
<tr><td>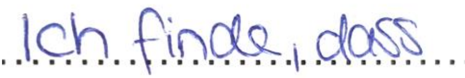
</td><td>Diese Linien sind für Ihre eigene Antwort gedacht. Schreiben Sie bitte möglichst leserlich.</td></tr>
<tr><td>☐ Sonstiges, und zwar:
……………………………</td><td>Bei diesem Hinweis können Sie zusätzlich eine eigene Antwort angeben.</td></tr>
</table>

Fragebogen

1. **Ich wohne seit ……………… *(Jahreszahl eintragen)* in der Gemeinde [redacted].**

2. **Ich wohne in einem:**
(Bitte nur ein Kreuz!)

☐ Einfamilienhaus
☐ Zweifamilienhaus
☐ Mehrfamilienhaus
☐ Seniorenheim
☐ Sonstiges, und zwar:
………………………….

3. **Mit mir im selben Haus wohnt/wohnen:**
(Mehrfachnennungen möglich!)

☐ Mein(e) (Ehe)Partner(in)
☐ Mein(e) Kind(er)
☐ Mein(e) Enkelkind(er)
☐ Mein(e) Geschwister
☐ Meine Eltern
☐ Andere Verwandte
☐ Freunde/-innen,
☐ Nachbarn/-innen Bekannte
☐ Meine(e) Haustiere(e)
☐ Niemand
☐ Sonstiges, und zwar:
…………………………

4. **Ich habe Kinder:**
(Bitte nur ein Kreuz!)

☐ Ja
☐ Nein

5. **Familienangehörige, die nicht mit mir im selben Haus, sondern außerhalb wohnen, treffe ich:**
(Bitte nur ein Kreuz!)

- ☐ (Mehrmals) täglich
- ☐ Wöchentlich
- ☐ Monatlich oder seltener
- ☐ Nie
- ☐ Ich habe keine

6. **Zu diesen Personen aus meinem <u>Familien- und Bekanntenkreis</u> habe ich 14-tägig normalerweise mindestens einmal Kontakt, d.h. ich führe mit ihnen ein <u>persönliches oder telefonisches Gespräch</u>:**
(Mehrfachnennungen möglich!)

- ☐ Ehe- oder Lebenspartner/-in
- ☐ Eigene Kinder
- ☐ Enkelkinder oder andere Verwandte
- ☐ Freunden/-innen/Bekannten/Vereinskollegen/-innen
- ☐ Nachbarn/-innen
- ☐ Niemand
- ☐ Sonstige, und zwar:

……………………………………………………………………

……………………………………………………………………

7. Zu diesen Personen aus dem <u>öffentlichen Bereich</u> habe ich 14-tägig normalerweise mindestens einmal Kontakt, d.h. ich führe mit ihnen ein <u>persönliches oder telefonisches Gespräch</u>:
(Mehrfachnennungen möglich!)

- ☐ Arzt/Ärztin
- ☐ Pflegekräfte/Mitarbeitende eines ambulanten Dienstes
- ☐ Pfarrer/-in oder Mitglieder einer Kirchengemeinde
- ☐ Friseur/-in
- ☐ Verkäufer/-in
- ☐ Niemand
- ☐ Sonstige, und zwar:

……………………………..………

……………………………...

8. Einsam fühle ich mich …
(Bitte nur ein Kreuz!)

- ☐ Selten oder nie
- ☐ Manchmal
- ☐ Oft

9. Ich möchte häufiger unter Leute kommen.
(Bitte nur ein Kreuz!)

- ☐ Stimme voll zu
- ☐ Stimme eher zu
- ☐ Stimmer eher nicht zu
- ☐ Stimme gar nicht zu

10. Meiner Meinung nach gibt es genügend gesellschaftliche/gesellige Angebote für Seniorinnen und Senioren in der Gemeinde [redacted]:
(Bitte nur ein Kreuz!)

☐ Ja ☐ Nein

11. Mit den aufgeführten Angeboten der Gemeinde [redacted] bin ich wie folgt zufrieden:

	Sehr zufrieden	Zufrieden	Unzufrieden	Sehr unzufrieden
Einkaufs-möglichkeiten	☐	☐	☐	☐
Freizeit-angebote	☐	☐	☐	☐
Kultur-angebote	☐	☐	☐	☐
Ärztliche Versorgung	☐	☐	☐	☐
Versorgung durch Apotheken	☐	☐	☐	☐
Notdienst der Ärzte	☐	☐	☐	☐
Notdienst der Apotheken	☐	☐	☐	☐
Öffentliche Verkehrs-mittel	☐	☐	☐	☐
Gaststätten	☐	☐	☐	☐

	Sehr zufrieden	Zufrieden	Unzufrieden	Sehr unzufrieden
Öffnungs-zeiten des Rathauses	□	□	□	□
Bisherige Angebote der Gemeinde, z.B. Senioren-nachmittage, -ausflüge, etc.	□	□	□	□
Sonstiges, und zwar: ……………. ……………. …………….	□	□	□	□

12. Folgende Angebote könnten mich dabei unterstützen, Kontakte zu pflegen oder neue Kontakte zu knüpfen:

	Stimme zu	Stimme eher zu	Stimme eher nicht zu	Stimme nicht zu
Essen in Gesellschaft	☐	☐	☐	☐
Gemeinsames Spazierengehen	☐	☐	☐	☐
Gemeinsames Musizieren, Singen	☐	☐	☐	☐
Tanzabende	☐	☐	☐	☐
Weiterbildungs-kurse (z.B. Fremd-sprachen, Umgang mit Internet oder Handy)	☐	☐	☐	☐
Unterstützungs-gruppe, in der man anderen Personen seine Hilfe anbieten und/oder Hilfe bekommen kann	☐	☐	☐	☐
Gemeinsame Bewegung/ Sport	☐	☐	☐	☐

	Stimme zu	Stimme eher zu	Stimme eher nicht zu	Stimme nicht zu
Gesellschafts-spiele spielen	☐	☐	☐	☐
Gemeinsames Kochen	☐	☐	☐	☐
Ausflüge	☐	☐	☐	☐
Informations-veranstaltungen zu verschiedenen Themen	☐	☐	☐	☐
Bastel-/ Malangebote	☐	☐	☐	☐
Gartenarbeit in Gesellschaft	☐	☐	☐	☐
Handwerkliche Tätigkeiten in Gesellschaft	☐	☐	☐	☐
Gottesdienst-besuche, Nutzen kirchlicher Angebote	☐	☐	☐	☐
Filmabende	☐	☐	☐	☐
Sonstige, und zwar: ………………..… ………………….. …………………..	☐	☐	☐	☐

13. Mein Gesundheitszustand im Allgemeinen ist:

(*Bitte nur ein Kreuz!)*

- ☐ Sehr gut
- ☐ Gut
- ☐ Mittelmäßig
- ☐ Schlecht
- ☐ Sehr schlecht

14. In meinem Alltag bin ich auf Geh- oder Mobilitätshilfen angewiesen:

- ☐ Nein
- ☐ Ja, und zwar auf:

  (Mehrfachnennungen möglich!)

  - ☐ Gehstock oder Gehilfen („Krücken“)
  - ☐ Rollator
  - ☐ Rollstuhl (mechanisch)
  - ☐ Rollstuhl (elektrisch)
  - ☐ Elektromobil für Senioren/-innen
  - ☐ Behindertengerechtes Auto
  - ☐ Sonstiges, und zwar:

    ....

15. Für gewöhnlich verlasse ich mein Haus/ meine Wohnung:
(Bitte nur ein Kreuz!)

- ☐ Täglich
- ☐ Mehrmals pro Woche, aber nicht täglich
- ☐ Einmal pro Woche, aber nicht häufiger
- ☐ Seltener als einmal pro Woche

16. Im Alltag verlasse ich mein Zuhause, um folgenden Erledigungen/Aktivitäten nachzugehen:

……………………………………………………………

……………………………………………………………

……………………………………………………………

17. Im Alltag hindern mich folgende Gegebenheiten/ Situationen daran, mein Zuhause für Erledigungen/Aktivitäten zu verlassen:

……………………………………………………………

……………………………………………………………

……………………………………………………………

18. Diese Verkehrsmittel benutze ich zur Erledigung der alltäglichen Besorgungen, wie z.B. Einkaufen, Arztbesuche, Apotheken, in der Freizeit, bei Kulturangeboten und dem Besuch von Freunden und Bekannten:

	Sehr häufig	Häufig	Gelegentlich	Nie
Ich gehe zu Fuß.	☐	☐	☐	☐
Fahrrad	☐	☐	☐	☐
Rollstuhl	☐	☐	☐	☐
Eigenes Auto	☐	☐	☐	☐
Ich werde von Anderen mitgenommen.	☐	☐	☐	☐
Öffentliche Verkehrsmittel	☐	☐	☐	☐
Taxi	☐	☐	☐	☐
Sonstige, und zwar: ………………... ………………... ………………...	☐	☐	☐	☐

19. Meine Freizeit gestalte ich mit:
(Mehrfachnennungen möglich!)

- ☐ Sportlicher Betätigung
- ☐ Spazierengehen
- ☐ Kurse besuchen/weiterbilden
- ☐ Gartenarbeit
- ☐ Ehrenamtlicher Tätigkeit
- ☐ Busreisen/Tagesausflüge
- ☐ Treffen mit Freunden/-innen/Bekannten
- ☐ Besuch kultureller Veranstaltungen
  (z.B. Theater, Kino, Museum, Konzerte)
- ☐ Vereinstätigkeit
- ☐ Zeitung/Bücher lesen
- ☐ Telefonieren
- ☐ Hausarbeit
- ☐ Betreuung von Familienangehörigen
  (z.B. Partner/in, Enkelkinder)
- ☐ Handwerklichen Tätigkeiten
- ☐ Basteln
- ☐ Grabpflege
- ☐ Sonstigem, und zwar:

  ……………………………………………………………

  ……………………………………………………………

20. Folgende Angebote würde ich gerne nutzen, wenn Sie hier in der Gemeinde [redacted] angeboten würden:

	Stimme zu	Stimme eher zu	Stimme eher nicht zu	Stimme nicht zu
Essen in Gesellschaft	☐	☐	☐	☐
Gemeinsames Spazierengehen	☐	☐	☐	☐
Gemeinsames Musizieren, Singen	☐	☐	☐	☐
Tanzabende	☐	☐	☐	☐
Weiterbildungs-kurse (z.B. Fremd-sprachen, Umgang mit Internet oder Handy)	☐	☐	☐	☐
Unterstützungs-gruppe, in der man anderen Personen seine Hilfe anbieten und/oder Hilfe bekommen kann	☐	☐	☐	☐
Gemeinsame Bewegung/ Sport	☐	☐	☐	☐

	Stimme zu	Stimme eher zu	Stimme eher nicht zu	Stimme nicht zu
Gesellschafts-spiele	☐	☐	☐	☐
Gemeinsames Kochen	☐	☐	☐	☐
Ausflüge	☐	☐	☐	☐
Informations-veranstaltungen zu verschiedenen Themen	☐	☐	☐	☐
Bastel-/ Malangebote	☐	☐	☐	☐
Gartenarbeit in Gesellschaft	☐	☐	☐	☐
Handwerkliche Tätigkeiten in Gesellschaft	☐	☐	☐	☐
Gottesdienst-besuche, Nutzen kirchlicher Angebote	☐	☐	☐	☐
Filmabende	☐	☐	☐	☐
Sonstige, und zwar: ………………….. ………………….. …………………..	☐	☐	☐	☐

Aus statistischen Gründen benötigen wir folgende Angaben zu Ihrer Person:

21. Ich wurde im Jahr 19………… *(Jahreszahl eintragen)* **geboren.**

22. Mein Geschlecht ist:
(Bitte nur ein Kreuz!)

□ Männlich □ Weiblich □ Divers

23. Insgesamt besuchte ich für ………….. Jahre die Schule, Hochschule oder eine andere schulische Ausbildung (ohne betriebliche Ausbildung).

24. Mein Erwerbsstatus beschreibt sich am besten mit: *(Mehrfachnennungen möglich!)*

□ Ich bin in Vollzeit erwerbstätig.
□ Ich bin in Teilzeit erwerbstätig.
□ Ich bin Hausfrau/-mann.
□ Ich arbeite in Altersteilzeit.
□ Ich bin im Vorruhestand.
□ Ich bin im Ruhestand/in Rente.
□ Ich gehe einem Minijob/450-Euro-Job nach.
□ Ich bin arbeitssuchend.
□ Sonstiges, und zwar:

…………………………………………………………

25. Die Bezeichnung der Tätigkeit/des Berufes, in welchem ich zuletzt tätig war, bzw. tätig bin (bei aktueller Erwerbstätigkeit) lautet:

………………………………………………………………

………………………………………………………………

26. Derzeit beziehe ich staatliche Leistungen:

- ☐ Nein
- ☐ Ja, und zwar:
  (Mehrfachnennungen möglich!)
  - ☐ Rente der gesetzlichen Rentenversicherung
  - ☐ Pension
  - ☐ Betriebliche oder öffentliche Zusatzversorgung
  - ☐ Grundsicherung im Alter/Sozialhilfe
  - ☐ Wohngeld
  - ☐ Leistungen der Pflegeversicherung
  - ☐ Sonstige, und zwar: ……………………………

27. Alle monatlichen Einkünfte in meinem Haushalt zusammengenommen, bleibt nach Abzug von Steuern und Beiträgen zur Renten-, Kranken-, Pflege- und Arbeitslosen-versicherung noch folgender Betrag übrig:
(Bitte nur ein Kreuz!)

- ☐ Bis unter 900€
- ☐ 900 bis unter 1.300€
- ☐ 1.300€ bis unter 1.500€
- ☐ 1.500€ bis unter 2.000€
- ☐ 2.000€ bis unter 2.600€
- ☐ 2.600€ bis unter 3.200€
- ☐ 3.200€ bis unter 4.500€
- ☐ 4.500€ und mehr

In meinem Haushalt leben, mich selbst eingerechnet, …………….. Personen *(Anzahl eintragen)***.**

28. Sonstige Anregungen oder Wünsche möchte ich noch mitteilen:

……………………………………………………………

……………………………………………………………

……………………………………………………………

……………………………………………………………

……………………………………………………………

……………………………………………………………

Herzlichen Dank für Ihre Teilnahme!
