# Supplementary material for: Offers to prevent social isolation and loneliness in older people living in rural areas: A community-based survey of needs
Source: Pravent Gesundh. 2023 Mar 15:1–10. [Article in German] Online ahead of print. doi: 10.1007/s11553-023-01025-8 (PMC10015520; doi:10.1007/s11553-023-01025-8)
Supplement: Supplementary file 2 — Anschreiben zum Fragebogen [file 11553_2023_1025_MOESM2_ESM.pdf]

**Gemeinde [redacted] 1. Bürgermeister, [redacted], Gesundheitsbotschafterin**
**[redacted], Studentin**

Sehr geehrte Damen und Herren,

schön, dass Sie sich Zeit für unseren Fragebogen nehmen.
Die Gemeinde [redacted] möchte in nächster Zeit insbesondere für Seniorinnen und Senioren neue Angebote schaffen, damit Sie auch in Zukunft gesund und gerne hier leben. Wir möchten nun von Ihnen wissen, was Sie bewegt und welche Angebote Sie interessieren könnten.
Bei Bedarf dürfen Sie sich Unterstützung beim Lesen oder Schreiben durch Angehörige oder Freunde einholen. Wichtig ist jedoch, dass Sie den Fragebogen nach Ihrer persönlichen Einschätzung ausfüllen. **Die Teilnahme ist freiwillig und anonym, alle Daten werden vertraulich behandelt.**
Wenn Sie den Fragebogen ausgefüllt und in den beigelegten Briefumschlag mit der Beschriftung „**Fragebogen**" gesteckt haben, gibt es drei Möglichkeiten, ihn bis zum **29.01.** auf den Weg zur Auswertung zu bringen:

1.) **Abgabe bei den folgenden Personen:**

[redacted]

- [redacted]; auch vor/nach den Gottesdiensten)

[redacted]

- Rathaus [redacted] (Briefkasten oder persönlich) ([redacted])
- Bürgermeister [redacted] ([redacted])

[redacted]

2.) **Abholung der Fragebögen durch das Gemeindepersonal:**
Wir bitten um telefonische Anforderung während der Öffnungszeiten im Rathaus [redacted] (Tel. [redacted]).

3.) **Einwurf in bereitstehende Sammelboxen:**

- Metzgerei [redacted]
- Bäckerei [redacted]
- Informationsveranstaltung in [redacted]/Gemeinschaftshaus am 29.01.2020

Als Dankeschön für Ihre Teilnahme an der Befragung können Sie mit der beigefügten Teilnehmererklärung an einem Gewinnspiel teilnehmen. Verlost werden 20 Gutscheine im Wert von je 10€, welche im Rahmen von Seniorenveranstaltungen der Gemeinde [redacted], welche über das Jahr 2020 stattfinden, für Speisen und Getränke eingelöst werden können. Eine Barauszahlung ist nicht möglich.

Die Verlosung der Preise erfolgt am 25.03.2020 im Rahmen einer Veranstaltung im Gemeinschaftshaus [redacted].

Haben Sie Fragen oder wünschen sich Unterstützung beim Ausfüllen des Fragebogens, so wenden Sie sich gerne während der Öffnungszeiten an das Rathaus [redacted] (Tel.: [redacted]).

Herzlichen Dank für Ihre Teilnahme an der Befragung!

Mit freundlichen Grüßen

[redacted]*, Bürgermeister der Gemeinde* [redacted]

[redacted]*, Gesundheitsbotschafterin der Gemeinde* [redacted]

[redacted]*, Studentin an der* [redacted]*; Durchführung und Auswertung der Befragung*

**Extra Blatt:**

**Teilnahme am Gewinnspiel**

Wenn Sie am Gewinnspiel teilnehmen möchten, füllen Sie bitte diesen Abschnitt aus und stecken Sie ihn in den Briefumschlag mit der Beschriftung „**Gewinnspiel“**. Geben Sie diesen genau wie den Fragebogen, aber in den **getrennten Briefumschlägen**, ab.
Die Teilnahme am Gewinnspiel ist **freiwillig**. Wer nicht am Gewinnspiel teilnehmen möchte, kann trotzdem an der Befragung teilnehmen. Die Daten werden **nicht** mit den Angaben auf dem Fragebogen in Verbindung gebracht.

**-------------------------------------------------------------------------------------------**

**O** Ja, ich möchte am Gewinnspiel teilnehmen. Für diesen Zweck gebe ich hier meinen Namen und meine Adresse an:

____________________________________________

*(Vorname, Nachname)*

____________________________________________

*(Straße, Hausnummer)*

____________________________________________

*(Ortsteil)*
